# Predictors of cost-effectiveness of selected COPD treatments in primary care: UNLOCK study protocol

**DOI:** 10.1038/npjpcrm.2015.51

**Published:** 2015-08-06

**Authors:** Job F M van Boven, Miguel Román-Rodríguez, Janwillem W H Kocks, Joan B Soriano, Maarten J Postma, Thys van der Molen

**Affiliations:** 1 Unit of PharmacoEpidemiology and PharmacoEconomics (PE2), Department of Pharmacy, University of Groningen, Groningen, The Netherlands; 2 Institituto de Investigacíon Sanitaria de Palma (IdISPa), Palma de Mallorca, Spain; 3 Department of General Practice, University Medical Centre Groningen, University of Groningen, Groningen, The Netherlands; 4 Instituto de Investigación Hospital Universitario de la Princesa (IISP), Universidad Autónonoma de Madrid, Cátedra UAM-Linde, Madrid, Spain; 5 Institute of Science in Healthy Aging and HealthcaRE (SHARE), University Medical Centre Groningen, University of Groningen, Groningen, The Netherlands

## Background

Chronic obstructive pulmonary disease (COPD) puts a high burden on patients and governmental health-care budgets.^1,2^ General practitioners (GPs) have a pivotal role in the treatment of COPD patients in primary care. However, the strategies of treatment may differ considerably between individual GPs, resulting in large intra-individual differences in health-care utilisation and quality of life of their patients.^3,4^ Recently, the Spanish AUDIPOC study showed great variability in hospital treatment patterns and patients’ outcomes.^5^ Moreover, the European COPD audit indicated marked differences in resources available across different hospitals in Europe.^6^ In Spain though, it is estimated that at least 61% of COPD patients are only treated in primary care,^7^ with an average of 6.6 visits per year. The estimated prevalence of COPD in the Balearic Islands is 12.8%.^8^ Regarding health-care costs for respiratory patients, several cost drivers, mostly related to patient characteristics, have been identified in previous studies including associated comorbidities (e.g., heart disease), forced expiratory volume in 1 s (FEV_1_), the physical component of quality of life, 6-min walking distance, increased dyspnoea, number of medical visits and hospitalisations.^9–11^ Although one study identified an effect of the individual physician on health-care costs,^12^ treatment strategies were never incorporated as a predicting variable for costs or outcomes. Besides inter-physician differences in treatments, country-specific regulations and difference in the extent of adherence to clinical guidelines may affect the cost-effectiveness of treating COPD patients in primary care settings.^13^ It was shown that adherence to COPD treatment guidelines is suboptimal.^14^ Moreover, non-adherence to guidelines was associated with higher total health-care costs.^15^ In particular, in times of increasing health-care costs and scarcer resources, there is a need to identify the cost-effectiveness of different treatment strategies for COPD patients across various primary care settings. The UNLOCK project of the International Primary Care Respiratory Group (IPCRG) offers a promising possibility.^16^

## Aims

The primary aim of this study is to assess what makes one COPD treatment strategy more cost-effective than others, by taking into account factors related to patients, the physician, and specific follow up and treatment approaches. A secondary objective is to assess whether real-world cost-effectiveness of treatments is comparable between Spain and other countries that have comparable data sets available.

## Methods

### Study design

This is a cost-effectiveness analysis that is performed with a real-world database on respiratory patients.

### Setting

This study comprises two phases, with the first phase including all primary care centres in the Balearic Islands, Spain. In a second phase of the study, primary care centres from other parts of the world will be included.

### Data source

All the data will be extracted from the MAJOrca Real-world Investigation in COPD and Asthma database (MAJORICA). The MAJORICA database contains combined data from the primary care system (e-SIAP), the hospital claims system (FIC), and the pharmacy database (RELE) in the Balearics, Spain. Together, these databases cover all health-care utilisation of the permanent inhabitants of the Balearics (±1.1 million subjects). In the Balearics, there are about 400 different GPs, and most of the COPD patients are treated by one of these GPs. The MAJORICA database contains data from all patients aged ⩾18 years with a primary care diagnosis of asthma and/or COPD in 2012, regardless of health insurance. All demographics, clinical data, diagnostic tests, as well as resource use, pharmacy dispense data, work absence and patient-reported outcomes from almost 70,000 respiratory patients are available for the period 2011–2014. A specification of the database is provided in Table 1. The database characteristics were reported according to the checklists of the IPCRG^16^ and the Respiratory Effectiveness Group (http://www.effectivenessevaluation.org). The unique island setting of the Balearics allows us to provide an almost complete picture of the real-world health-care use of COPD patients.

### Inclusion criteria

All patients (⩾18 years) with a clinical diagnosis of COPD (ICD-9 codes: 491, 492, 496 and/or primary codes R79, R95) in 2012, available in the MAJORICA database, were included. In addition, patients needed to be a permanent resident of the Balearic Islands and to be alive in 2014.

### Health-care resource utilisation

Health-care resource use in 2013 and 2014 will be calculated for all the COPD patients identified in 2012. Health-care resource use that will be included in the study refers to the following: GP visits, primary care nurses visits, emergency department (ED) visits, specialist visits, specialist nurse visits, hospitalisations, medication and diagnostic tests (that is, spirometry, CT-scans, X-rays, bronchoscopy). To estimate indirect costs, data on work absence will be extracted. These data will be extracted from the e-SIAP system, as work absence in Spain is registered by GPs.

### Calculation of health-care costs and indirect costs

Total costs will be calculated by multiplying each unit of resource use and lost workdays with standard cost-per-unit prices, which are obtained from the Health Care Administration Office of the Balearics.^17^

### Predictors for cost-effectiveness

Predictors for cost-effective treatment will be assessed, including variables related to patient, physician or treatment. Predictors related to patients may include age, gender, body mass index, smoking status, exacerbations (physician diagnosis and/or prescription of prednisone), COPD severity by spirometry, short-acting β_2_-agonist use, health-related quality of life and comorbidity. Examples of predictors related to the physician are age, gender and setting, number of patients per practice and number of COPD patients per practice. Predictors related to treatment may include prescription of medication and adherence based on refill of medication, influenza vaccination in the past year, requests for diagnostic tests, referrals to hospital or specialists and the use of patient-reported outcomes (PROs).

### Comparisons

Specifications of the comparisons that could potentially be made, depending on the exact data available, are listed in Table 2.

### Data analysis

The total patients’ sample will be split into two groups, depending on the treatment variables that will be compared (Table 2). For example, to assess the impact of using PROs, all patients who were treated by a GP who uses PROs will be selected as the treatment group. An equal group of control patients, not treated by a GP who uses PROs, will be selected using a matching procedure. The matching procedure (based on propensity scores) will use patient characteristics (age, gender, smoking status) and disease severity (FEV_1_, exacerbations, quality of life, comorbidities).

For both groups, the average total costs per patient (as well as minimum, maximum and standard deviation) will be calculated on the basis of the direct health-care costs, as listed above (hospitalisations, medication, ED visits), and indirect costs. The cost difference between the two groups will result in a ∆C variable to obtain an estimate of the incremental costs. The differences in effect size (∆E) will be expressed as the difference in health effects between the two groups that are compared. The health effects depend on what variables will be consistently available in the database. Exacerbations avoided will be used, as well as changes in COPD-specific changes in the quality of life, as defined by the COPD Assessment Test (CAT) or modified Medical Research Council (mMRC) questionnaire.

Subsequently, the incremental cost-effectiveness ratio (ICER) can be calculated as follows: (Costs_group1_−Costs_group2_)−(Effects_group1_−Effects_group2_)=∆C/∆E, which provides the incremental costs per exacerbation avoided or incremental costs per CAT point gained. The ICER will be calculated using both the health-care payer’s and the societal perspective. The societal perspective includes work productivity costs. Sensitivity analyses will be performed using the minimal and maximal costs (scenario analyses), as well as a bootstrap procedure (as patient-level data will be available). Bootstrapping relies on random sampling with replacement, and it will allow estimating accuracy (such as 95% confidence intervals) to sample estimates.

### External validity using UNLOCK

Once the predictors have been identified, we will invite members of the UNLOCK project in other countries (e.g., The Netherlands, Sweden and others) to participate to test the external validity and inter-country variation of these predictors.

To assure consistency of the analytic process and consequent results, data will be compared with other data sets from different IPCRG countries, including the same variables and applying the same methods.

### Ethical approval

Ethical approval was granted by the local primary care research committee.

## Discussion

Current clinical treatment guidelines are mainly based on evidence from large clinical trials with a selective study population, which does not seem to reflect the majority of patients treated in real-world primary care.^18,19^ Therefore, there is an urgent need to assess the validity of treatment recommendations when applied in real-world treatment. Results from this study are expected to provide useful insights in the cost-effectiveness of the broad range of strategies and factors related to the primary care treatment of COPD. The use of a real-world database that covers the complete Balearic population is considered a major strength, as a representative population is assessed in which the risk of pre-selection bias is limited. A second strength is that results will be compared with other international settings, thereby increasing generalisability. Here, the UNLOCK project of IPCRG offers a useful possibility.^16^ However, given the retrospective observational design, some limitations should be acknowledged. First, by the use of real-world data, missing data are common. In particular, registration of data regarding the use of spirometry, smoking status and patient-reported outcomes is expected to be limited. Pulmonary rehabilitation and physiotherapy data are not included in the effectiveness analysis because of the difficulty in collecting such data and because of the limited availability of these services. In addition, miscoding or incomplete and invalid data collection may have occurred because of the real-word setting. Another limitation lies in the observational design, which usually increases the risk for bias. Although the database itself covers the complete population, the individual analyses are prone to selection bias. To minimise this risk of bias, a matching procedure will be used, but unobserved bias may still occur. Despite these limitations, the need for more real-world evidence and comparative effectiveness research is increasing, thereby strengthening the overall relevance of this study.^20^

## Figures and Tables

**Table 1:** Specification of the MAJORICA database

*Variable*	*Specification*
*Type of database*	
Electronic Medical Record	Yes
Claims	Yes

Country/countries of data origin	Balearics, Spain
Number of patients	68,578
Patients with asthma diagnosis (ICD-9: 493)	45,800
Patients with COPD diagnosis (ICD-9: 491, 492, 496)	27,871
Patients with asthma and COPD diagnosis (ICD-9: 493 and (491. 492, 496))	5,093
Data collection (period)	2011–2014
Unique identifier/anonymisation	Yes
Ethical approval	Yes

*Coding system diseases*	
ICD-9, ICD-10, read	ICD-9

*Patient demographics*	
Gender	Yes
Age	Yes
BMI	Yes

*Physician demographics*	
Gender	Yes
Age	Yes
Setting (urban/rural)	Yes

*Drugs*	
Coding	ATC-7
Prescribed, dispensed, both	Dispensed
Drugs available	All R03
Dose/dosing	Yes
Device	No
OTC medications	No
Inhaler technique	No

*Vaccinations*	
Influenza, Pneumococcal	Yes

*Outcomes*	
Exacerbations	
Steroids	Yes
Antibiotics	Yes
SABA	Yes
Exacerbations (ICD-9 code)	Yes
Health resource utilisation	
Primary care consultations	Yes
Secondary care consultations	Yes
Consultations coded by disease	Yes
Consultations coded by routine/emergency	Yes
Hospitalisations	Yes
Hospitalisations coded by disease	Yes
Hospitalisation duration	Yes
Emergency room	Yes
ICU	Yes
ICU coded by disease	Yes
ICU duration	Yes
Rehabilitation	No
Physiotherapy	No
Patient-reported	
mMRC	Yes
Asthma (ACQ, ACT)	ACT score
COPD (CCQ, CAT)	CAT score
Side effects	
Pneumonias	Yes
Work absence	
All cause	Yes
Respiratory specific	Yes

*Covariates*	
Comorbidities	
Diabetes	Yes
Cardiovascular diseases	
Hypertension	Yes
Cardiac insufficiency	
Atrial fibrillation	
Cor pulmonale	
Allergic rhinitis	Yes
Cerebrovascular disease	Yes
Osteoporosis	Yes
Sleep apnoea	Yes
Nasal polyps	No
Depression/anxiety	Yes
Reflux (GERD)	Yes
Chronic kidney disease	Yes
Lung Cancer	Yes
AIDS/HIV	Yes
Cognitive dysfunction	No
Risk score	
Cardiovascular risk score	Yes
Lifestyle	
Smoking status	Yes
Smoking years	Yes
Socioeconomic status	
Post code	No
Education level	No
Employment status	Yes
Salary range	No
Spirometry	
FEV_1_/FVC, FEV_1_%pred, reversibility	Yes
Laboratory tests	
Full blood count, FeNO, IgE and so on	No
Imaging	
CRX	Performed Y/N
HRCT	Performed Y/N

Abbreviations: ACT, asthma control test; ATC, anatomical therapeutic chemical; BMI, body mass index; CAT, COPD Assessment Test; CRX, chest X-ray; FEV, forced expiratory volume; FVC, forced vital capacity; GERD, gastroesophageal reflux disease; HRCT, high-resolution computed tomography; ICD, International Classification of Diseases; ICU, intensive care unit; mMRC, modified Medical Research Council; N, no; OTC, over the counter; SABA, short-acting beta agonists; Y, yes.

**Table 2:** Comparison of cost-effectiveness to be potentially made between groups

*Predictors related to patient*		
Age	<75 years	75 years or more
Gender	Male	Female
BMI	<25	25 or more
Smoking status	Current smoker	Former smoker or non-smoker
Exacerbations	<2	2 or more
Hospitalisations	0	1 or more
Severity by FEV_1_	<50%	50% or more
Use of SABA	<2 dispenses per year	2 or more
Comorbidity	<2	2 or more
Cardiovascular	No	Yes
HRQoL	CAT<10	10 or more
GP visits	<2	2 or more
Medication adherence	<80%	80% or more

*Predictors related to GP*		
Age	<35	35 years or older
Gender	Male	Female
Region	Urban	Rural
Use of PROs	Yes	No
Requests for lab/tests	Yes	No

*Predictors related to specific treatment*		
Influenza vaccination	Yes	No
LABA	Yes	No
LAMA	Yes	No
LABA-ICS	Yes	No

Abbreviations: BMI, body mass index; CAT, COPD Assessment Test; GP, general practitioner; HRQoL, health-related quality of life; ICS, inhaled corticosteroid; LABA, long-acting β_2_-agonist; LAMA, long-acting muscarinic antagonist; PROs, patient-reported outcomes; SABA, short-acting β_2_-agonist.

## Notes

JWHK and JBS are Associate Editors of *npj Primary Care Respiratory Medicine*, but were not involved in the editorial review of, nor the decision to publish, this article. None of the other authors declare any conflict of interest.
